# *IL-7/IL-7R* gene variants impact circulating IL-7/IL-7R homeostasis and ART-associated immune recovery status

**DOI:** 10.1038/s41598-019-52025-8

**Published:** 2019-10-31

**Authors:** Andra Ceausu, Esther Rodríguez-Gallego, Joaquim Peraire, Miguel López-Dupla, Pere Domingo, Consuelo Viladés, Judit Vidal-Gonzalez, Maria Peraire, Carles Perpiñán, Yolanda María Pacheco, Sergi Veloso, Verónica Alba, Montserrat Vargas, Alfonso J. Castellano, Ezequiel Ruiz-Mateos, Josep Mallolas, Francesc Vidal, Anna Rull

**Affiliations:** 1 Hospital Universitari de Tarragona Joan XXIII, IISPV, Universitat Rovira i Virgili, Tarragona, Spain; 2 Infectious Diseases Unit, Hospital de la Santa Creu i Sant Pau, Barcelona, Spain; 3 Universitat de Barcelona, Barcelona, Spain; 4 Universitat Rovira i Virgili, Tarragona, Spain; 5 Laboratory of Immunology, Institute of Biomedicine of Seville, IBiS, UGC Clinical Laboratories, Virgen del Rocío University Hospital/CSIC/University of Seville, Seville, Spain; 6 Clinic Unit of Infectious Diseases, Microbiology and Preventive Medicine, Institute of Biomedicine of Seville, Virgen del Rocío University Hospital/CSIC/University of Seville, Seville, Spain; 7 HIV Unit. Infectious Diseases Service, Hospital Clinic, Universitat de Barcelona, Barcelona, Spain; 8 Present Address: Servei de Medicina Interna-Hepatologia, Hospital Universitari de la Vall d’Hebron, VHIR, Barcelona, Spain; 9 Present Address: Hospital Universitari Son Espases, Palma de Mallorca, Spain; 10 Present Address: Current address: Atenció Primària ICS, Cap Sant Pere, Reus, Spain

**Keywords:** Predictive markers, HIV infections

## Abstract

A relationship between polymorphisms in genes encoding interleukin 7 (IL-7) and its cellular receptor (IL-7R) and antiretroviral therapy (ART)-associated immune recovery in HIV subjects has been previously reported. However, details of this relationship remain unclear, and the association of these polymorphisms with circulating IL-7/IL-7R levels is scarce. Here, we explored whether IL-7/IL-7R axis was associated with quantitative CD4^+^ T-cell recovery in HIV-infected subjects. *IL-7/IL-7R* polymorphisms were assessed by genotyping, and multiple inheritance models were used to estimate both, their association with low pre-ART CD4^+^ T-cell counts and incomplete immune recovery status after 48 weeks of suppressive ART. Integrated data from genetic variants association and soluble plasma IL-7/IL-7R quantification suggest that IL-7/IL-7R genotype expression could alter the homeostatic balance between soluble and membrane-bound receptors. The haplotype analyses indicates that allele combinations impacts pre-ART circulating CD4^+^ T-cell counts, immune recovery status and the absolute increment of CD4^+^ T-cell counts. The knowledge about how IL-7/IL-7R axis is related to quantitative CD4^+^ T-cell recovery and immune recovery status after initiating ART could be useful regarding T-cell reservoirs investigations in HIV subjects.

## Introduction

Untreated HIV infection usually causes a progressive decrease in the number of circulating CD4^+^ T-cells over time. This may be due to underproduction and/or overdestruction of these cells^1^. The decrease is often profound enough to put a patient at risk of developing opportunistic infections that may ultimately lead to death. One of the objectives of antiretroviral treatment (ART) is to improve the prognosis of HIV-infected subjects due to the decrease of HIV replication below detectable levels and due to CD4^+^ T-cell count recovery, achieving cell counts that preclude patients from the risk of opportunistic infections. However, while this goal is achieved in a substantial proportion of patients, up to 30% of infected subjects who successfully suppress viremia below the limit of detectability do not obtain sufficient CD4^+^ T-cell gains^2^. These patients are known as “poor recoverers” or “nonrecoverers”^2,3^, and they have worse clinical outcomes compared with “good recoverers”^4–6^.

The forces that drive the CD4^+^ T-cell depletion and recovery dynamics are not fully known, but virus-, drug- and host-related factors may all be involved. Among host factors, perturbations in molecules involved in CD4^+^ T-cell homeostasis have been sought. In addition, cytokines have a key master regulatory role in T-cell homeostasis. Among them, interleukin 7 (IL-7) together with its cellular receptor (IL-7R) regulates thymic output of T cells^7,8^, promotes memory T-cell proliferation^9^, and mediates the survival of peripheral T-cells^10^. The importance of IL-7 in CD4^+^ T-cell turnover is highlighted by the fact that administration of recombinant human IL-7 increases CD4^+^ T-cell counts both in patients with idiopathic CD4^+^ lymphocytopenia^11^ and in HIV-infected subjects on ART, which, despite achieving virological suppression, do not fully recover CD4^+^ T-cell counts^12,13^.

Given the essential role of IL-7 in CD4^+^ T-cell homeostasis, some investigations have tried to explore the association between plasma IL-7 levels and CD4^+^ T-cell gain due to ART^14–16^ and the relationship between variations in genes encoding the IL-7/ IL-7R axis^17–20^. However, the results are still inconsistent, and investigations regarding the role of the IL-7/IL-7R axis are needed to understand the biologic mechanisms associated with nonrecoverers. Thus, in this study, we determined whether *IL-7* and *IL-7R* single nucleotide polymorphisms (SNPs) are associated with CD4^+^ T-cell recovery in ART-naïve HIV-infected subjects. IL-7 and IL-7R plasma levels were also evaluated in baseline samples of HIV-infected subjects who first began ART and again after 48 and 144 weeks of follow-up under the ART regimen.

## Results

### Patient characteristics

**Figure 1:**
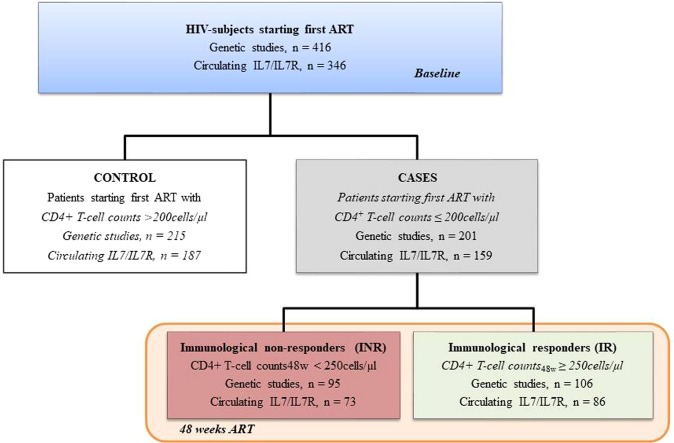
Flow chart illustrating subject cohort enrolment and analysis. HIV-infected subjects were included and categorized into controls and cases according to pre-ART CD4^+^ T-cell counts. For an immune recovery substudy group, cases starting ART with T-cell counts below 200 cells/µL were categorized according to their immune status after 48 weeks of follow-up.

The pre-ART clinical characteristics of the overall cohort of HIV-infected subjects (n = 416) categorized according to classification criteria (Fig. 1) are presented in Table 1. Immunological nonrecoverers (INR) subjects were older, presented significantly decreased CD4^+^ T-cell counts and increased plasma viral loads and were more likely to be diagnosed with prior AIDS-related illness. Pre-ART circulating plasma IL-7 and IL-7R values were also included for 346 samples and described below.

**Table 1:** Study cohort (n = 416) characteristics of the according classification criteria.

	CD4^+^ T-cells ≤200 cells/µL		CD4^+^ T-cells >200 cells/µL	P- value
	INR (n = 95)	IR (n = 106)	Control (n = 215)	
**Pre-ART clinical characteristics**				
Age at cART initiation (years)	42 [37–51]	37 [33–42]	37 [31–45]	**<0.001**
Male	58 (79)	70 (82)	155 (83)	0.807
Risk factor				*0.060*
Heterosexual	29 (40)	31 (36)	62 (33)	
Homo/Bisexual	26 (36)	47 (55)	101 (54)	
Intravenous drug abuse	15 (20)	6 (7)	19 (10)	
Other/Unknown	3 (4)	2 (2)	5 (3)	
CD4^+^ T-cell count (cells/µL)	51 [17–111]	129 [51–181]	333 [272–441]	**<0.001**
Plasma HIV RNA load (log copies/mL)	5.30 [4.82–5.62]	5.06 [4.71–5.61]	4.87 [4.33–5.25]	**<0.001**
AIDS indicator condition	30 (41)	21 (24)	13 (7)	**<0.001**
HCV co-infection (Positive)	13 (20)	10 (12)	27 (15)	0.410
**ART clinical characteristics**				
48 weeks CD4^+^ T-cell count (cells/µL)	167 [110–216]	350 [294–422]	532 [408–699]	**<0.001**
ΔCD4^+^ T-cell count	95 [47–142]	239 [187–316]	170 [64–289]	**<0.001**
**Pre-ART plasma IL-7/IL-7R (n** = **345)**	**(n** = **73)**	**(n** = **86)**	**(n** = **187)**	
Log IL-7 (pg/mL)	2.65 [2.53–2.82]	2.67 [2.54–2.81]	2.72 [2.60–2.99]	**0.037**
Log IL-7R (ng/mL)	2.34 [2.02–2.49]	2.33 [2.08–2.54]	2.34 [2.16–2.63]	0.264

Data are presented as n (%) or median (interquartile range). Categorical data were compared by means of a χ^2^ test, whereas continuous data were compared using non-parametric Kruskall-Wallis test. P value < 0.05 was considered significant and is highlighted in bold. All P values > 0.05 but <0.15 were considered relevant for results interpretation and are italicized. AIDS was diagnosed according the CDC1993 criteria. INR, incomplete immune recoverers; IR, immune recoverers.

### Genetic association study for *IL-7* gene variants

Figure S1 summarizes allele and genotype frequencies for *IL-7* gene variants located on chromosome 8, which were in accordance with data listed on the NCBI SNP database. The genotype frequencies for *rs6987789* and *rs7007634* were consistent with HWE (Figure S1-B). After verifying that there was no association between these *IL-7* gene variants and low pre-ART CD4^+^ T-cell counts (CD4^+^ T-cell ≤ 200 cells/μL) (Figure S1-C, cases versus controls), we investigated whether there was any association with poor ART-associated immune response (Figure S1-C, INR versus immunological recoverers (IRs)). No association was found between these *IL-7* gene variants and incomplete immune recovery status. Finally, in the multiple-SNP analysis, linkage disequilibrium (LD) was found between *rs6987789* and *rs7007634* (D’ = 0.2713, r = 0.0982, P = 0.055). Any of the possible haplotypes were related to ART-associated immune recovery (Table S1).

### Association between *rs10491434 (IL-7R)* and low pre-ART CD4^+^ T-cell counts

Figure S2 summarizes allele frequencies for *IL-7R* gene variants on chromosome 5, which were in accordance with data listed on the NCBI SNP database. The genotype frequencies for the *IL-7R* gene variants explored in this study were consistent with HWE, except for *rs969128* (Table S2). Then, we searched for an association between the *IL-7R* gene variants and low pre-ART CD4^+^ T-cell counts. Considering Akaike’s Information Criteria (AIC) and Bayesian Information Criteria (BIC) scores and adjusting for age and baseline pre-ART CD4^+^ T-cell counts, an association was only detected with the overdominant model for *rs10491434* (Table S3). In the multiple-SNP analysis (Figure S3 and Table S4), LD was found between several genetic variants (Figure S3), and the haplotype TAGAGCTCTAAT, which is present in 16% of the study cohort, was related to low pre-ART CD4^+^ T-cell counts (Table S4-B).

### Two haplotypes from the *IL-7R* gene variants associated with immune restoration

**Figure 2:**
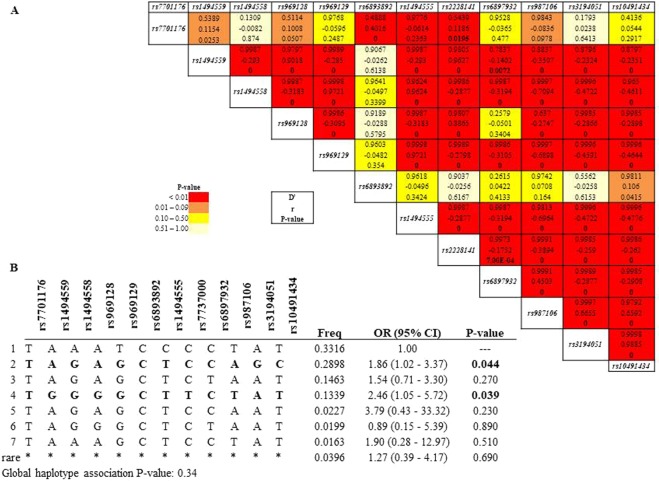
Haplotype analysis for the *IL-7R* gene variants explored in this study. (**A**) Linkage disequilibrium (LD) analysis in INR subjects compared to IR subjects for *IL-7R* gene variants. (**B**) Haplotype association with immune recovery status according to CD4^+^ T-cell counts after 48 weeks of ART (n = 197).

Next, we explored the association between *IL-7R* gene variants and immune recovery status. Considering AIC and BIC scores and adjusting for age and baseline pre-ART CD4^+^ T-cell counts, the best associations were with the recessive models for *rs1494558* (OR = 0.16, 95% CI = 0.05–0.57, P = 0.0017), *rs969129* (OR = 0.29, 95% CI = 0.09–0.91, P = 0.025) and *rs1494555* (OR = 0.20, 95% CI = 0.06–0.67, P = 0.0044) (Table 2). LD was found between several gene variants (Fig. 2), and the haplotypes TAGAGCTCCAGC (OR = 1.86, 95% CI = 1.02–3.37, P = 0.04) and TGGGGCTTCTAT (OR = 2.46, 95% CI = 1.05–5.72, P = 0.039) were associated with the immune response according to CD4^+^ T-cell counts after 48 weeks of ART (Table S5, Fig. 2).

**Table 2:** *IL-7R* SNP association with poor immune recovery after 48 weeks of cART.

Polymorphism	IHT model	Genotype	INR	IR	OR (95% CI)	P-value	AIC	BIC
rs7701176 T > A	—	T/T	91 (97.8%)	104 (100%)	1.00	0.083	230.1	243.2
		T/A	2 (2.1%)	0 (0%)	0.00 (0.00-NA)			
rs1494559 A > G	Dominant	A/A	66 (77.7%)	68 (68.7%)	1.00	0.420	220.7	233.6
		G/A-G/G	19 (22.4%)	31 (31.3%)	1.35 (0.65–2.83)			
rs1494558 G > A	Recessive	G/G-G/A	68 (81%)	94 (95.9%)	1.00	**0.002**	205.8	218.6
		A/A	16 (19.1%)	4 (4.1%)	0.16 (0.05–0.57)			
rs969128 A > G	Dominant	A/A	64 (79%)	70 (68%)	1.00	0.240	211.3	224.2
		G/A-G/G	17 (21%)	33 (32%)	1.57 (0.74–3.36)			
rs969129 G > T	Recessive	G/G-T/G	71 (82.6%)	93 (94.9%)	1.00	**0.025**	214	226.9
		T/T	15 (17.4%)	5 (5.1%)	0.29 (0.09–0.91)			
rs6893892 C > T	—	C/C	92 (98.9%)	100 (99%)	1.00	0.970	231.5	244.6
		T/C	1 (1.1%)	1 (1%)	1.07 (0.06–18.78)			
r1494555 T > C	Recessive	T/T-C/T	70 (80.5%)	90 (94.7%)	1.00	**0.004**	203	215.9
		C/C	17 (19.5%)	5 (5.3%)	0.20 (0.06–0.67)			
rs2228141 C > T	Dominant	C/C	72 (80%)	68 (68%)	1.00	0.250	224	237
		T/C-T/T	18 (20%)	32 (32%)	1.54 (0.73–3.22)			
rs6897932 C > T	Recessive	C/C-T/C	81 (94.2%)	99 (99%)	1.00	0.082	221.5	234.4
		T/T	5 (5.8%)	1 (1%)	0.17 (0.02–1.63)			
rs987106 T > A	Dominant	T/T	31 (34.1%)	24 (23.8%)	1.00	0.140	225.9	238.9
		T/A-A/A	60 (65.9%)	77 (76.2%)	1.69 (0.84–3.43)			
rs3194051 A > G	Dominant	A/A	47 (53.4%)	46 (46.5%)	1.00	0.200	217	229.9
		G/A-G/G	41 (46.6%)	53 (53.5%)	1.54 (0.79–2.99)			
rs10491434 T > C	Dominant	T/T	46 (53.5%)	45 (45.9%)	1.00	0.150	213.9	226.8
		T/C-C/C	40 (46.5%)	53 (54.1%)	1.63 (0.83–3.18)			

The logistic regression model was adjusted by age and pre-ART CD4^+^ T-cell counts. Data analysis is summarized with n (%), odds ratio (OR) and 95% confidence interval (CI) for each SNP. P value of the likehood ratio test less than <0.05 was considered significant and is highlighted in bold. The AIC and BIC values were used to choose the inheritance model (IHT) that best fits the data. AIC; Akaike’s Information Criteria; BIC; Bayesian Information Criteria.

### Association between circulating IL-7 values and pre-ART CD4^+^ T-cell counts

Pre-ART circulating IL-7/IL-7R values were evaluated in 346 plasma samples, including 187 controls and 159 cases (73 INRs and 86 IRs). The cases revealed lower circulating IL-7 values than the controls [2.66 [2.53–2.81] log pg/mL and 2.72 [2.60–2.98] log pg/mL, respectively, P = 0.03]. In fact, correlation analysis indicated a positive association between circulating IL-7 values and low pre-ART CD4^+^ T-cell counts (ρ = 0.106, P = 0.045). No differences were observed in IL-7R values. However, IL-7R values were significantly associated with circulating IL-7 values (ρ = 0.692, P < 0.001), a correlation that was consistent in the cases (ρ = 0.683, P < 0.001) and the controls (ρ = 0.693, P < 0.001).

Then, we explored whether changes in the IL-7/IL-7R axis could predict ART-associated immune recovery status. No differences in pre-ART circulating IL-7/IL-7R values were observed between INRs and IRs (Table 1). When correlation analyses were performed among the different groups, we observed a positive association between IL-7R values and the ΔCD4^+^ T-cell counts (ρ = 0.246, P = 0.023) in IRs.

### *IL-7R* gene variants impact circulating IL-7R in IRs

**Figure 3:**
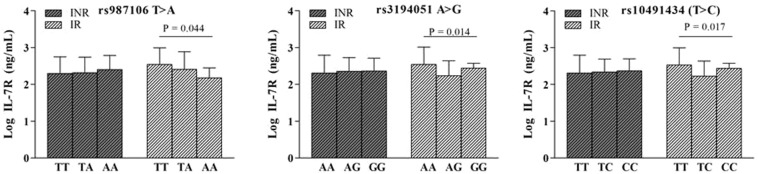
Influence of *IL-7R* genes in circulating concentrations of IL-7R. *Rs987106*, *rs3194051* and *rs10491434* variants influence circulating plasma concentrations in IR subjects. Data are represented as the mean ± SD (65 INRs and 75 IRs). The relationship between *IL-7R* genes and plasma IL-7 concentrations were analyzed using one-way ANOVA test (SPSS software).

The plasma IL-7/IL-7R axis was not influenced by any of the *IL-7* and *IL-7R* SNPs analyzed. However, when the impact of *IL-7/IL-7R* gene variants was explored in INRs and IRs individually, IRs revealed a significant association between circulating plasma IL-7R concentrations and *rs987106, rs3194051*, and *rs10491434* (Fig. 3). A strong LD among these *IL-7R* SNPs (Fig. 2, D’ > 0.95) was found, meaning that these alleles of each gene are inherited together more often than would be expected by chance.

### rs3194051 (IL-7R) influences the absolute increment of CD4+ T-cell counts

**Figure 4:**
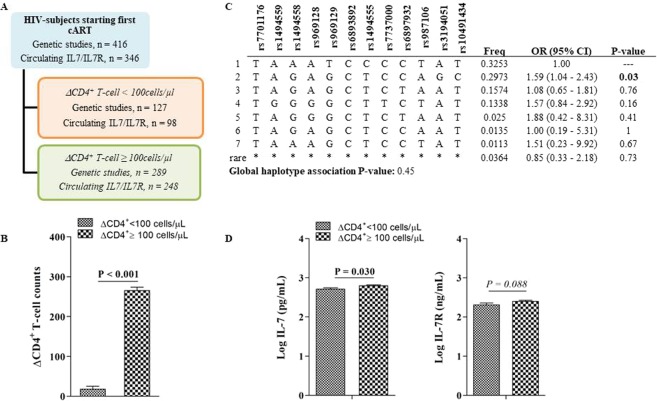
Impact of IL-7/IL-7R axis on the absolute increment of CD4+ T-cell counts. (**A**) Flow chart illustrating classification criteria according the absolute increment of CD4+ T-cell counts (ΔCD4+ T-cell) after 48 weeks of ART. (**B**) ΔCD4^+^ T-cell count mean ± SD values in cases2 and controls2 according to this classification criterion (n = 416). (**C**) Haplotype association with the ΔCD4+ T-cell count (n = 452). (**D**) Pre-ART circulating concentrations of IL-7 (n = 356) and IL-7R (n = 349) according ΔCD4+ T-cell count criteria. Data are represented as the mean ± SD. Comparisons between groups were performed with nonparametric Mann-Whitney (MW) (SPSS software).

Because the ΔCD4^+^ T-cell count was significantly lower in INRs (Table 1), we also decided to verify any possible association between the ΔCD4^+^ T-cell count and the IL-7/IL-7R axis. Therefore, all subjects (n = 416) were reclassified in two groups according ΔCD4^+^ T-cell count after 48 weeks of ART (Fig. 4A), and those with a ΔCD4^+^ T-cell count <100 cells/µL (n = 136) were considered poor immune recoverers and were clearly differentiated from immune recoverers (Fig. 4B).

First, no association was found between *IL-7* gene variants and ΔCD4^+^ T-cell counts. Any of the possible haplotypes were related to these absolute increments (global P-value = 0.44, Table S6). However, regarding the *IL-7R* gene variants, the best associations were with the recessive models for *rs3194051* (OR = 2.51, 95% CI = 0.97–6.48, P = 0.04) (Table S7). LD was found between several gene variants (data not shown), and the haplotype TAGAGCTCCAGC, which was previously associated with immune response (Fig. 2), was also associated with a ΔCD4^+^ T-cell count >100 cells/μL (Fig. 4C).

Second, according to this classification criterion, subjects with ΔCD4^+^ T-cell counts <100 cells/μL after 48 weeks of ART regimen revealed decreased circulating IL-7/IL-7R values (Fig. 4D).

### Longitudinal evaluation of IL-7/IL-7R plasma concentrations

**Figure 5:**
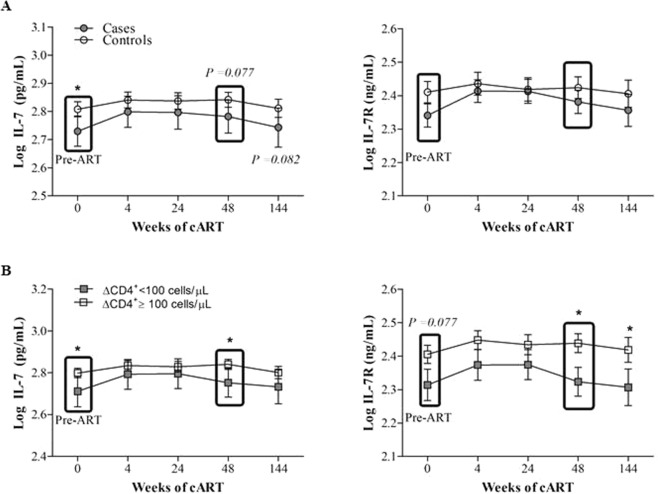
Longitudinal evaluation of the *IL-7/IL-7R* axis during 144 weeks of ART. (**A**) Longitudinal study of circulating IL-7 and IL-7R values in the cases and the controls during 144 weeks of ART. (**B**) Longitudinal study of circulating IL-7 and IL-7R values in subjects classified according to the ΔCD4+ T-cell count criterion during 144 weeks of ART. Data are represented as the mean ± SEM Comparisons between groups were performed with nonparametric Mann-Whitney (MW) tests for unpaired samples and a Wilcoxon t-test for paired samples (W) (SPSS software).

First, we examined plasma IL-7/IL-7R concentrations at different time points during the 48 weeks of the ART regimen. We also included data from 197 HIV-infected subjects with available follow-up samples after 144 weeks on ART. Circulating IL-7 concentrations remained lower in the cases than the controls during the 144 weeks of ART. By contrast, IL-7R values became similar until 48 weeks of ART, when IL-7R values were again lower in the cases than the controls (Fig. 5A). No differences were observed among INRs and IRs (data not shown).

Finally, we evaluated the association between IL-7/IL-7R parameters and ΔCD4^+^ T-cell. Circulating IL-7 concentrations remained lower in subjects with a ΔCD4^+^ T-cell count <100 cells/µL, with significant differences at 48 weeks of ART. In addition, circulating IL-7R values also remained lower in subjects with a ΔCD4^+^ T-cell counts <100 cells/µL, and these differences were also significant after 48 and 144 weeks of ART (Fig. 5B).

## Discussion

This is the first study to assess both the association of IL-7 genetic background and circulating IL-7 behavior overtime with quantitative immune recovery in treated HIV-infected patients. Some previous evidence suggests that genetic variation within the IL-7/IL-7R axis may modulate the magnitude of CD4^+^ T-cell gains^17–20^, although the underlying mechanisms remain unclear. On the other hand, whereas an increase in IL-7 production has been proposed as part of the homeostatic response to T-cell depletion^16^ and thus relates lower pre-ART IL-7 levels to faster CD4^+^ T cell recovery^18^, other studies suggest an association between lower concentrations of the IL-7/IL-7R axis with an insufficient ability to restore the number of CD4^+^ T cells in response to ART^14,21^. In this longitudinal study, we demonstrated that IL-7R gene variants were associated with low pre-ART CD4^+^ T cell counts and immune restoration according to immune recovery status after 48 weeks on ART. Furthermore, our data revealed that IL-7R gene variants impact pre-ART IL-7R plasma concentrations and the absolute increment of CD4^+^ T cell counts during ART. Circulating pre-ART IL-7 levels were low in HIV-infected subjects with low pre-ART CD4^+^ T cell counts and significantly correlated with pre-ART IL-7R plasma concentrations. In addition, we observed that IL-7/IL-7R values remained lower in subjects with an increment of CD4^+^ T-cell counts of less than 100 CD4^+^ T-cells/μL during the follow-up until 144 weeks of ART than in those with ΔCD4^+^ T-cell counts greater than 100 cells/µL. To our knowledge, this study is the first description of the relationship between IL-7/IL-7R polymorphisms and pre-ART IL-7/IL-7R plasma concentration in relation to low pre-ART CD4^+^ T-cell counts and immune restoration due to ART.

First, no association was found between IL-7 gene variants and low pre-ART CD4^+^ T-cell counts, immune restoration or absolute increment of CD4^+^ T-cell counts due to ART. Similar to our data, in a previous case-controlled association study exploring the influence of the IL-7/IL-7R pathway on HIV-1 pathogenesis and AIDS progression, no association with IL-7 gene variants could be found^22^. Notice that IL-7 is a nonredundant cytokine that is crucial for B- and T-cell homeostasis that mediates a plenitude of functions in health and disease^23^. Therefore, its importance for the survival and development and proliferation of B- and T-cells could explain the low levels of IL-7 gene diversity^24^, which was also previously observed in multiple sclerosis (MS)^25^.

Second, the *rs10491434* variant of the IL-7R gene and the H3 haplotype tagged by *rs10491434*-T (17% controls versus 14% cases) were associated with pre-ART CD4^+^ T-cell counts. *rs10491434* has been previously implicated in AIDS progression^22^, and the *rs10491434* T/T genotype is associated with HIV subjects that achieve CD4^+^ T-cell counts higher than 500 CD4^+^ T-cells/μL after 48 months on ART^26^. Consistent with this, in our study, patients with *rs10491434* T/T-C/C genotypes were more closely associated with high pre-ART CD4^+^ T-cell counts (CD4^+^ T-cells >200 cells/μL) before initiating ART therapy than were patients with *rs10491434* T/C genotypes. Notably, *rs10491434* is located at the 3′ UTR, showing allele-specific methylation at nearby CpG sites^27^; more specifically, the *rs10491434* T allele associated with lower methylation at nearby CpG sites increases IL-7R expression^26^. In fact, in our study, we found a significant association between *rs10491434* and circulating pre-ART IL-7R values in IRs. Concretely, IR subjects with the *rs10491434* TT genotype (major allele homozygous) showed higher plasma IL-7R values than did patients with the *rs10491434* TC/CC genotype. Additionally, circulating IL-7R values revealed a positive correlation with the absolute increment of CD4^+^ T-cell counts in this subgroup of subjects, corroborating the association between this gene variant and CD4^+^ T-cell recovery. Additionally, this 3′ gene region polymorphism is in LD with several SNPs that could impact the function of the IL-7 receptor, among which are the intronic SNP *rs987106* and the exonic SNP *rs3194051*. In this sense, not only the *rs10491434* genotype impacts circulating IL-7R concentrations in IRs but also the *rs987106* and *rs319405* genotypes seem to influence pre-ART circulating IL-7R concentrations. In the case of the *rs987106* gene variant, IRs carrying the T-allele revealed higher IL-7R values compared with those of the patients carrying the A-allele, in accordance with previous data^19^. *rs987106-*T was previously associated with the prevention of rapid HIV progression^22^, corroborating our findings that suggest that the intronic variant *rs987106-*T could be involved in CD4^+^ T-cell restoration and thus to facilitate disease evolution. Regarding the *rs3194051* gene variant, a dual role has been identified. First, the AA genotype was related to higher pre-ART IL-7R values in IRs, and the H4 haplotype tagged by *rs3194051*-A was associated with immune recovery status (15.5% in IRs versus 10.3% in INRs), also suggesting protecting effect of this polymorphism. In agreement with this conclusion, allele G was previously found to be associated with susceptibility to MS, a chronic autoimmune T-cell mediated disease of the central nervous system in which T-cells play an important role^28–30^. Therefore, *rs3194051-G* SNP could potentiate an inflammatory status which is one of the main characteristic of MS risk, whereas *rs3194051-A* could confer some anti-inflammatory properties to IRs compared to INRs which are at higher risk of developing opportunistic infections. On the other hand, our results also indicated a recessive genetic effect (GG versus GA + AA) relating the G allele to the absolute increment of more than 100 CD4^+^ T-cells/μL. In accordance with this IHT model, the H2 haplotype tagged by *rs3194051*-G, which is more frequent than the aforementioned H4 haplotype tagged by *rs3194051*-A, was associated with both successful immune recovery status (31.7% in IRs versus 27.4% INRs) and a ΔCD4^+^ T-cell count of more than 100 cells/μL (31% in ΔCD4^+^ ≥100 cells/μL versus 26% ΔCD4^+^ <100 cells/μL). Consistent with these data, homozygous carriers of the G-allele at *rs3194051* experienced faster time to CD4^+^ T-cell count >500 cells/μL compared with that of homozygous carriers of the A-allele subjects who were recruited from the Uganda AIDS Rural Treatment Outcomes (UARTO) cohort^19^. Additionally, HIV/HCV coinfected patients with the *rs3194051* AA genotype showed a higher probability of severe liver fibrosis than did patients with the *rs3194051* AG/GG genotype. The haplotype tagged by *rs3194051*-A had higher odds of having advanced liver fibrosis than did the G haplotype HIV/HCV coinfected patients^31^, suggesting an association between the A allele and worse disease prognosis. Furthermore, the dissimilar genotype distributions of *rs3194051* are evidence of the modulation of the IL-7/IL-7R axis during ART and its role in the expression of the subpopulation of T-cells.

Moreover, regarding IL-7R gene variants, we detected CD4^+^ T-cell restoration after ART therapy in HIV subjects carrying the haplotypes tagged by *rs1494558-*G, *rs969129-*G and *rs1494555-*T (major allele of each SNP). Notice that these three IL-7R polymorphisms were previously related to each other^31,32^, which is not surprising due to the strong LD. Consistent with our data, *rs1494558*-A was related to new-onset diabetes development after transplantation, playing an important role in the homeostasis of Treg cells, the regulatory T cells that play a major role in controlling immune responses to self-antigens^33^. Notice that diabetes is a metabolic complication associated with increased cardiovascular disease risk, a non-AIDS-defining clinical event, and INRs are associated with worse long-term clinical prognosis, including a higher risk of progression toward non-AIDS-defining clinical events^34^. Later, the *rs1494558*-A and *rs1494555*-C genotypes were also associated with an increased risk of acute and chronic graft versus host disease after allogeneic hematopoietic cell transplantation^35^. In our study, haplotypes tagged by *rs969129-*G and *rs1494555-*T were related to immune restoration due to ART. In the case of *rs14494555*, consistent with our data, a haplotype tagged by *rs1494555-*C was referred to as the ‘risk’ haplotype because it was more prominent in MS patients compared with healthy donors, whereas the opposite effect has been observed for other haplotypes tagged by *rs1494555-*T, which has therefore been termed the ‘protective’ haplotype^36^.

Our study has some limitations. First, the number of patients per groups in genetic studies is not consistent with the number of available samples for the study of circulating plasma IL-7/IL-7R concentrations, which would make our results more consistent in the search of predictive and diseases progression markers. Additionally, specific data regarding the ART therapy would have been helpful to evaluate the implication of the different anti-retroviral drugs on circulating IL-7/IL-7R axis. Nevertheless, previous evidences suggest no differences between different drug families and IL-7/IL-7R axis.

In conclusion, taken together, our data suggest that IL-7/IL-7R mRNA expression and genotype could alter the homeostatic balance between soluble and membrane-bound receptors. We corroborate that IL-7R polymorphisms could affect T-cell homeostasis and function and that some IL-7R polymorphisms due to their position could influence other IL-7R polymorphisms, and combinations could impact immune pathologies. Further investigation of the IL-7/IL-7R axis could also elucidate new insights regarding T-cell reservoirs in HIV subjects.

## Patients and Methods

### Study design and participants

This was a multicentered, longitudinal case-controlled study comprising 416 adult HIV-infected subjects who were consecutively recruited between 2011 and 2013 at the HIV outpatient clinic of the participating hospitals and who started their first ART and achieved virological suppression after ART. Patients were selected from among those who were receiving a combination of two nucleoside reverse transcriptase inhibitors (NRTI) plus a non-nucleoside reverse transcriptase inhibitor (NNRTIs) or a protease inhibitor(s) (PI). A flow chart with patient selection and enrolment is provided in Fig. 1 and inclusion/exclusion criteria defined in Supplementary material. Of the selected patients, 215 were controls (baseline CD4^+^ T-cell counts >200 cells/μL), and 201 were cases (baseline CD4^+^ T-cell counts ≤200 cells/μL). Among the cases, 106 subjects achieved more than 250 CD4^+^ T-cells/µL after 48 weeks of ART (“immunological recoverers”, IRs), and 95 subjects did not reach the 250 cells/µL CD4^+^ T-cell threshold (“immunological nonrecoverers”, INRs). The absolute increment of CD4^+^ T-cell counts was also calculated to re-classify HIV subjects into those that achieved more than 100 CD4^+^ T-cell counts/µL after 48 of ART and those that did not. The study and all research protocols were carried out in accordance with the recommendations of the Ethical and Scientific Committees from each participating institution (Hospital Universitari de Tarragona Joan XXIII (Tarragona), Hospital de la Santa Creu i Sant Pau (Barcelona), Virgen del Rocío University Hospital (Seville), Hospital Clinic (Barcelona)) and were approved by the Committee for Ethical Clinical Research following the rules of Good Clinical Practice from the Institut d’Investigació Sanitària Pere Virgili (CEIm IISPV). The CEIm IISPV is an independent committee, made up of health and non-health professionals, which supervises the correct compliance of the ethical principles governing clinical trials and research projects that are carried out in our environment, specifically in its methodology, ethics and laws. All subjects gave written informed consent in accordance with the Declaration of Helsinki.

### General laboratory measurements

Blood was drawn from a peripheral vein after an overnight fast. Whole blood was used to determine the CD4^+^ T-cell count and for DNA isolation. Plasma was obtained by centrifugation and was stored at −80 °C until use. HIV-1 infection was diagnosed by a positive ELISA and confirmed by Western blot analysis. Plasma HIV-1 viral load was determined by the Cobas Amplicor HIV-1 Monitor Test v 1.5 (Roche Diagnostics, Barcelona, Spain). The limit of detectability is <20 copies/µL. CD4^+^ T-cell counts were analyzed using a flow cytometer FAC Scan (Becton Dickinson, San Jose, CA, USA).

### Genetic studies

We selected 14 single nucleotide polymorphisms (SNPs) for *IL7* and *IL7R* genes, with an allelic frequency greater than 20% in the Iberian Population in Spain (IBS) listed on the NCBI SNP database. In summary, the 14 SNPs analyzed in this study were the following for *IL-7: rs6987789* and *rs7007634;* and for *IL-7R: rs7701176, rs1494559, rs1494558, rs969128, rs969129, rs6893892, rs1494555, rs2228141 (rs7737000), rs6897932, rs987106, rs3194051* and *rs10491434*.

Genomic DNA was extracted from peripheral blood with Qiagen kit (Qiagen, Hilden, Germany) and then the extracted DNA samples (5 ng/μL) were sent to LGC Genomics Ltd. (formerly Kbioscience Ltd., Herts, UK) for genotyping. The number of individuals (n = 416) to be included in the genetic studies was at least 90 per group (Figs 1 and 4).

### IL-7 and IL-7R plasma concentrations

Plasma concentrations of human IL-7 (log pg/mL) and IL-7R (log ng/mL) were measured by double-antibody sandwich one-step process enzyme-linked immunosorbent assay (ELISA) QY-E04239 and QY-E04260, respectively (QAYEE-BIO, Shanghai, China), according to the manufacturer’s instructions (Supplementary material).

### Statistical analyses

Prior to the statistical analyses, the normal distribution and homogeneity of the variances were tested using a Kolmogorov-Smirnov test. Normally distributed data were expressed as the mean ± standard deviation (SD), whereas variables with a skewed distribution were represented as the median (25^th^ percentile–75^th^ percentile) or transformed into a decimal logarithm. Categorical variables were reported by number (percentages). Qualitative variables were analyzed using the χ2 test or Fisher’s exact test when as necessary. Comparisons between groups were performed with nonparametric Kruskal-Wallis (KW) and/or Mann-Whitney (MW) tests for unpaired samples and a Wilcoxon t-test for paired samples (W). Associations between quantitative variables were evaluated using the Spearman correlation. Allele and genotype frequencies and Hardy-Weinberg equilibrium (HWE) were evaluated using SNPstats software^37^. To estimate the association between *IL-7* and *IL-7R* genetic polymorphisms and immune recovery status, we use multiple inheritance models (codominant, dominant, recessive, overdominant and additive). For each SNP, odds ratios (ORs) and 95% confidence intervals (CIs) were calculated using unconditional logistic regression analysis with adjustment of age and baseline pre-cART CD4+ T-cell counts. Statistical analyses were performed using SPSS (version 21.0, SPSS Inc., Chicago, IL), and graphical representations were generated with GraphPad Prism software (version 5.0, GraphPad Inc., San Diego, CA). The results were considered significant at P < 0.05.

## Supplementary information

Supplementary info

## Data Availability

The datasets used and/or analyzed during the current study are available from the corresponding author on reasonable request.
