# Supplementary material for: IL-7/IL-7R gene variants impact circulating IL-7/IL-7R homeostasis and ART-associated immune recovery status
Source: Sci Rep. 2019 Oct 31;9:15722. doi: 10.1038/s41598-019-52025-8 (PMC6823538; doi:10.1038/s41598-019-52025-8)
Supplement: Supplementary file 1 — Supplementary info [file 41598_2019_52025_MOESM1_ESM.pdf]

Supplementary Data for

IL-7/IL-7R gene variants impact circulating IL-7/IL-7R homeostasis and ART-associated immune recovery status

Andra Ceausu[1+], Esther Rodríguez-Gallego[1+], Joaquim Peraire[1], Miguel López-Dupla[1], Pere Domingo[2], Consuelo Viladés[1], Judit Vidal-Gonzalez[3a], Maria Peraire[4b], Carles Perpiñán[4c], Yolanda María Pacheco[5], Sergi Veloso[1], Verónica Alba[1], Montserrat Vargas[1], Alfonso J Castellano[1], Ezequiel Ruiz-Mateos[6], Josep Mallolas[7], Francesc Vidal[1++,*], Anna Rull[1++]

[1]Hospital Universitari de Tarragona Joan XXIII, IISPV, Universitat Rovira i Virgili, Tarragona, Spain
[2]Infectious Diseases Unit, Hospital de la Santa Creu i Sant Pau, Barcelona, Spain
[3]Universitat de Barcelona, Barcelona, Spain
[4]Universitat Rovira i Virgili, Tarragona, Spain
[5]Laboratory of Immunology, Institute of Biomedicine of Seville, IBiS, UGC Clinical Laboratories, Virgen del Rocío University Hospital/CSIC/University of Seville, Seville, Spain
[6]Clinic Unit of Infectious Diseases, Microbiology and Preventive Medicine, Institute of Biomedicine of Seville, Virgen del Rocío University Hospital/CSIC/University of Seville, Seville, Spain
[7] HIV Unit. Infectious Diseases Service, Hospital Clinic, Universitat de Barcelona, Barcelona, Spain

+ These authors contributed equally to this paper, and both should be considered as primary co-authors.
++ These authors contributed equally to this paper, and should both be considered as senior co-authors.

aCurrent address: Servei de Medicina Interna-Hepatologia, Hospital Universitari de la Vall d'Hebron, VHIR, Barcelona, Spain.
bCurrent address: Hospital Universitari Son Espases, Palma de Mallorca, Spain.
cCurrent address: Atenció Primària ICS, Cap Sant Pere, Reus, Spain.
*Correspondence to fvidalmarsal.hj23.ics@gencat.cat

Supplementary Material

Supplementary Tables

Table S1. Haplotype analysis for the *IL-7* gene variants explored in cases (INR versus IR).

Table S2. Basic information of the *IL-7R* gene variants in the overall study cohort.

Table S3. *IL-7R* SNP association with low pre-ART $CD4^+$ T-cell counts adjusted by age and baseline $CD4^+$ T-cell counts.

Table S4. Haplotype analysis for the *IL-7R* gene variants explored in this study.

Table S5. Haplotype frequencies estimation in INR compared to IR (n = 201).

Table S6. Genetic study for the *IL-7* gene variants in subjects categorized according the absolute increment of CD4+ T-cell counts after 48 weeks of ART.

Table S7. *IL-7R* SNP association with the absolute increment of $CD4^+$ T-cell counts after 48 weeks of ART.

Supplementary Figures

Figure S1. Genetic study for IL-7 gene variants.

Figure S2. Summary of allele frequencies for *IL-7R* single nucleotide polymorphisms (SNPs) in this study and in the Iberian Population in Spain (IBS) listed on the NCBI SNP database.

Figure S3. Linkage disequilibrium analysis in cases (low pre-ART $CD4^+$ T-cell counts) versus controls for *IL-7R* gene variants.

Supplementary Material

Inclusion/Exclusion criteria

All the selected patients had to fulfil the following inclusion criteria: age over 18 years, presence of HIV-1 infection, on ART regimen during the 48 weeks of the study and undetectable plasma HIV-1 viral load at 48 weeks. The exclusion criteria were the presence of active opportunistic infections, current inflammatory diseases or conditions, changes in ART regimen and alternative plasma C reactive protein >1 mg/dL.

Study design

The absolute increment of CD4+ T-cell counts using the formula: CD4+ T-cell counts at 48 weeks of ART - CD4+ T-cell count at baseline, before ART therapy.

Genetic studies

The sample size was calculated to find a difference in the distribution of polymorphisms between the two subsets of 13%, a risk alpha of 5% and a power of 80%.

IL-7 and IL-7R plasma concentrations

The assay range for IL-7 was 15.6 pg/mL - 1000 pg/mL, with an intra-assay variation coefficient of 5 pg/mL and an interassay variation coefficient of 3.24 pg/mL. For IL-7R, the assay range was 7.8 ng/mL - 1000 ng/mL, with an intra-assay variation coefficient of 6 ng/mL and an interassay variation coefficient of 4.38 ng/mL.

Table S1. Haplotype analysis for the *IL-7* gene variants explored in cases (INR versus IR). **A**) Haplotype frequencies estimation in INR compared to IR (n = 200) and **B**) haplotype association with immune recovery status according $CD4^+$ T-cell counts after 48 weeks of ART. The logistic regression model was adjusted by age and baseline (pre-ART) $CD4^+$ T-cell counts (n = 196).

A

	**rs6987789**	**rs7007634**	**Total**	**Cases**	**Controls**
1	G	A	0.9033	0.8905	0.9164
2	G	G	0.0842	0.0989	0.0693
3	T	A	0.0083	0	0.0143
4	T	G	0.0042	0.0105	NA

B

	**rs6987789**	**rs7007634**	**Freq**	**OR (95% CI)**	**P-value**
1	G	A	0.9038	1.00	---
2	G	G	0.0834	0.80 (0.33 - 1.94)	0.62
rare	*	*	0.0128	2.29 (0.28 - 18.45)	0.44

Global haplotype association P-value: 0.63

Table S2. Basic information of the *IL-7R* gene variants in the overall study cohort.

Polymorphism	Genotype	Cases		HWE	Controls		HWE	INR		HWE	IR		HWE
		Count	Proportion		Count	Proportion		Count	Proportion		Count	Proportion	
rs7701176 T>A	T/T	198	0.99	1	211	0.99	1	93	0.98	1	105	0.99	1
	T/A	3	0.01		3	0.01		2	0.02		1	0.01	
rs1494559 A>G	A/A	137	0.73	0.54	151	0.74	0.56	68	0.78	1	69	0.68	0.46
	A/G	49	0.26		47	0.23		18	0.21		31	0.31	
	G/G	2	0.01		5	0.02		1	0.01		1	0.01	
rs1494558 G>A	G/G	79	0.42	0.87	91	0.46	0.26	34	0.40	0.26	45	0.45	0.09
	G/A	86	0.46		80	0.41		36	0.42		50	0.50	
	A/A	21	0.11		25	0.13		16	0.19		5	0.05	
rs969128 A>G	A/A	137	0.73	**0.012**	146	0.74	**<0.0001**	66	0.80	0.086	71	0.68	0.09
	A/G	41	0.22		36	0.18		14	0.17		27	0.26	
	G/G	10	0.05		16	0.08		3	0.04		7	0.07	
rs969129 A>G	G/G	84	0.45	0.87	100	0.49	0.078	36	0.41	0.26	48	0.48	0.33
	G/T	82	0.44		78	0.38		36	0.41		46	0.46	
	T/T	21	0.11		27	0.13		15	0.17		6	0.06	
rs6893892 C>T	C/C	196	0.99	1	208	0.99	1	94	0.99	1	102	0.99	1
	C/T	2	0.01		3	0.01		1	0.01		1	0.01	
rs1494555 T>C	T/T	81	0.44	0.75	95	0.48	0.11	35	0.39	0.27	46	0.47	0.33
	T/C	82	0.44		78	0.39		37	0.42		45	0.46	
	C/C	23	0.12		27	0.14		17	0.19		6	0.06	
rs2228141 C>T	C/C	143	0.74	0.54	153	0.75	0.55	74	0.80	1	69	0.68	0.29
	C/T	49	0.25		46	0.23		17	0.18		32	0.31	
	T/T	2	0.01		5	0.02		1	0.01		1	0.01	
rs6897932 C>T	C/C	134	0.71	0.16	135	0.65	0.13	64	0.73	0.05	70	0.69	0.46
	C/T	50	0.26		60	0.29		19	0.22		31	0.30	
	T/T	6	0.03		12	0.06		5	0.06		1	0.01	
rs987106 T>A	T/T	56	0.29	0.15	57	0.27	0.07	31	0.33	0.10	25	0.24	0.84
	T/A	88	0.45		91	0.44		38	0.41		50	0.49	
	A/A	52	0.27		60	0.29		24	0.26		28	0.27	
rs3194051 A>G	A/A	94	0.49	1	182	0.50	0.51	47	0.52	0.79	47	0.47	0.82
	A/G	80	0.42		82	0.40		35	0.39		45	0.45	
	G/G	17	0.09		21	0.10		8	0.09		9	0.09	
rs10491434 T>C	T/T	92	0.49	0.86	104	0.50	0.19	46	0.52	0.44	46	0.46	0.82
	T/C	78	0.41		80	0.38		33	0.38		45	0.45	
	C/C	18	0.10		24	0.12		9	0.10		9	0.09	

HWE, Hardy-Weinberg equilibrium.

Table S3. *IL-7R* SNP association with low pre-ART CD4$^+$ T-cell counts adjusted by age and baseline CD4$^+$ T-cell counts.

**Polymorphism**	**IHT model**	**Genotype**	**Cases**	**Controls**	**OR (95% CI)**	**P-value**	**AIC**	**BIC**
rs7701176 T>A	---	T/T<br>T/A	195 (99%)<br>2 (1%)	208 (98.6%)<br>3 (1.4%)	1.00<br>0.63 (0.00-NA)	NA	26.2	42.2
rs1494559 A>G	Dominant	A/A<br>G/A-G/G	134 (72.8%)<br>50 (27.2%)	148 (74%)<br>52 (26%)	1.00<br>1.26 (0.07-24.32)	0.88	25.6	41.4
rs1494558 G>A	Dominant	G/G<br>G/A-A/A	78 (42.9%)<br>104 (57.1%)	90 (46.6%)<br>103 (53.4%)	1.00<br>0.34 (0.01-10.10)	0.52	21	36.7
rs969128 A>G	Dominant	A/A<br>G/A-G/G	134 (72.8%)<br>50 (27.2%)	143 (73.3%)<br>52 (26.7%)	1.00<br>0.41 (0.01-19.21)	0.64	24.3	40.1
rs969129 G>T	Dominant	G/G<br>T/G-T/T	83 (45.1%)<br>101 (54.9%)	99 (49%)<br>103 (51%)	1.00<br>0.11 (0.00-3.22)	0.15	21.1	36.9
rs6893892 C>T	---	C/C<br>T/C	192 (99%)<br>2 (1%)	205 (98.6%)<br>3 (1.4%)	1.00<br>NA (0.00-NA)	0.74	25.9	41.9
r1494555 T>C	Dominant	T/T<br>C/T-C/C	80 (44%)<br>102 (56%)	94 (47.7%)<br>103 (52.3%)	1.00<br>0.43 (0.02-7.49)	0.56	24.1	39.9
rs2228141 C>T	Dominant	C/C<br>T/C-T/T	140 (73.7%)<br>50 (26.3%)	150 (74.6%)<br>51 (25.4%)	1.00<br>1.25 (0.07-23.97)	0.88	26	41.9
rs6897932 C>T	Dominant	C/C<br>T/C-T/T	130 (69.9%)<br>56 (30.1%)	133 (65.2%)<br>71 (34.8%)	1.00<br>13.42 (0.20-891.35)	0.16	20.9	36.7
rs987106 T>A	Dominant	T/T<br>T/A-A/A	55 (28.6%)<br>137 (71.3%)	55 (26.8%)<br>150 (73.2%)	1.00<br>2.69 (0.06-114.35)	0.60	25.9	41.8
rs3194051 A>G	Overdominant	A/A-G/G<br>G/A	110 (58.8%)<br>77 (41.2%)	121 (59.9%)<br>81 (40.1%)	1.00<br>0.10 (0.00-5.46)	0.18	21.3	37.2
rs10491434 T>C	Overdominant	T/T-C/C<br>T/C	109 (59.2%)<br>75 (40.8%)	126 (61.5%)<br>79 (38.5%)	1.00<br>0.00 (0.00-2.99)	**0.01**	16.5	32.4

Data analysis is summarized with n (%), odds ratio (OR) and 95% confidence interval (CI). The AIC and BIC values were used to choose the inheritance model (IHT) that best fits the data. HWE, Hardy-Weinberg equilibrium; AIC, Akaike's Information Criteria; BIC, Bayesian Information Criteria.

Table S4. Haplotype analysis for the *IL-7R* gene variants explored in this study. **A**) Haplotype frequencies estimation in cases compared to controls (n = 416) and **B**) haplotype association with low pre-ART CD4$^+$ T-cell counts (n =409). The logistic regression model was adjusted by age and baseline pre-ART CD4$^+$ T-cell counts.

A

	rs7701176	rs1494559	rs1494558	rs969128	rs969129	rs6893892	rs1494555	rs7737000	rs6897932	rs987106	rs3194051	rs10491434	Total	Cases	Controls
1	T	A	A	A	T	C	C	C	C	T	A	T	0.3253	0.3375	0.3162
2	T	A	G	A	G	C	T	C	C	A	G	C	0.297	0.2916	0.2997
3	T	A	G	A	G	C	T	C	T	A	A	T	0.1573	0.144	0.1704
4	T	G	G	G	G	C	T	T	C	T	A	T	0.134	0.1314	0.1377
5	T	A	G	G	G	C	T	C	T	A	A	T	0.0252	0.0188	0.0298
6	T	A	G	A	G	C	T	C	C	A	A	T	0.0136	0.023	0.005
7	T	A	A	A	G	C	T	C	C	T	A	T	0.0111	0.016	0.0058
8	Other haplotypes that are in a proportion less than 0.01 in all the cohort														

B

	rs7701176	rs1494559	rs1494558	rs969128	rs969129	rs6893892	rs1494555	rs7737000	rs6897932	rs987106	rs3194051	rs10491434	Freq	OR (95% CI)	P-value
1	T	A	A	A	T	C	C	C	C	T	A	T	0.321	1.00	---
2	T	A	G	A	G	C	T	C	C	A	G	C	0.2965	7.97 (0.54 - 117.67)	0.13
3	**T**	**A**	**G**	**A**	**G**	**C**	**T**	**C**	**T**	**A**	**A**	**T**	0.1595	1300.21 (20.46 - 82635.08)	**0.0008**
4	T	G	G	G	G	C	T	T	C	T	A	T	0.1366	141.94 (0.25 - 82076.09)	0.13
5	T	A	G	G	G	C	T	C	T	A	A	T	0.0253	34.66 (0.00 - 8369829.54)	0.58
6	T	A	G	A	G	C	T	C	C	A	A	T	0.0139	2175.87 (0.03 - 183701217.77)	0.18
7	T	A	A	A	G	C	T	C	C	T	A	T	0.0113	30.87 (0.00 - 13185924.45)	0.60
rare	*	*	*	*	*	*	*	*	*	*	*	*	0.0359	311.96 (0.06 - 1602960.17)	0.19

Global haplotype association P-value: 0.77

Table S5. Haplotype frequencies estimation in INR compared to IR (n = 201).

	rs7701176	rs1494559	rs1494558	rs969128	rs969129	rs6893892	rs1494555	rs7737000	rs6897932	rs987106	rs3194051	rs10491434	Total	INR	IR
1	T	A	A	A	T	C	C	C	C	T	A	T	0.337	0.400	0.273
2	T	A	G	A	G	C	T	C	C	A	G	C	0.292	0.274	0.317
3	T	A	G	A	G	C	T	C	T	A	A	T	0.144	0.148	0.141
4	T	G	G	G	G	C	T	T	C	T	A	T	0.131	0.103	0.155
5	T	A	G	A	G	C	T	C	C	A	A	T	0.023	0.016	0.029
6	T	A	G	G	G	C	T	C	T	A	A	T	0.019	0.017	0.020
7	T	A	A	A	G	C	T	C	C	T	A	T	0.016	0.012	0.019
8	Other haplotypes that are in a proportion less than 0.01 in all the cohort														

Table S6. Genetic study for the *IL-7* gene variants in subjects categorized according the absolute increment of CD4+ T-cell counts after 48 weeks of ART. **A**) *IL-7* SNP association with absolute increment of CD4+ T-cell counts adjusted by age and baseline $CD4^+$ T-cell counts (n = 409). **B**) Haplotype frequencies estimation and **B**) haplotype association with absolute increment of CD4+ T-cell counts after 48 weeks of ART. The logistic regression model was adjusted by age and baseline (pre-ART) $CD4^+$ T-cell counts (n = 414). *Cumulative frequency.

A

Polymorphism	IHT model	Genotype	<100 cells/µL	≥100 cells/µL	OR (95% CI)	P-value	AIC	BIC
rs6987789 G>T	---	G/G	124 (99.2)	276 (97.2)	1.00	0.28	546.1	770.9
		T/G	1 (0.8)	8 (2.8)	2.97 (0.33-26.90)			
rs7007634 A>G	Overdominant	A/A	103 (84.4)	225 (80.9)	1.00	0.28	529.1	752.6
		G/A-G/G	19 (15.6)	53 (19.1)	1.45 (0.73-2.86)			

B

	rs6987789	rs7007634	Total	$\Delta CD4^+$ T-cell counts <100 cells/µL	≥100 cells/µL	Frequency*
1	G	A	0.8932	0.9141	0.8839	0.8932
2	G	G	0.0958	0.082	0.102	0.9890
3	T	A	0.0092	0	0.0136	0.9982
4	T	G	0.0018	0.004	0.0005	1

	rs6987789	rs7007634	Freq	OR (95% CI)	P-value
1	G	A	0.8933	1.00	---
2	G	G	0.0957	1.27 (0.67 - 2.44)	0.47
rare	*	*	0.011	2.99 (0.33 - 27.20)	0.33

Table S7. *IL-7R* SNP association with the absolute increment of $CD4^+$ T-cell counts after 48 weeks of ART. The model was adjusted by age and baseline $CD4^+$ T-cell counts, and the IHT model selected considering AIC and BIC scores.

Polymorphism	IHT model	Genotype	<100 cells/µL	≥100 cells/µL	OR (95% CI)	P-value	AIC	BIC
rs7701176 T>A	---	T/T	125 (98.4)	284 (98.6)	1.00	0.49	553.9	779.4
		T/A	2 (1.6)	4 (1.4)	0.51 (0.08-3.22)			
rs1494559 A>G	Dominant	A/A	93 (80.9)	195 (70.7)	1.00	0.10	509.9	732.2
		G/A-G/G	22 (19.1)	81 (29.4)	1.66 (0.90-3.07)			
rs1494558 G>A	Recessive	G/G-G/A	95 (82.6)	241 (90.3)	1.00	0.07	510.5	727.5
		A/A	20 (17.4)	26 (9.7)	0.50 (0.24-1.05)			
rs969128 A>G	Dominant	A/A	91 (81.2)	192 (70.1)	1.00	0.09	504.8	718.4
		G/A-G/G	21 (18.8)	82 (29.9)	1.70 (0.92-3.15)			
rs969129 G>T	Dominant	G/G	52 (43.7)	132 (48.4)	1.00	0.27	515.6	738.0
		T/G-T/T	67 (56.3)	141 (51.6)	0.75 (0.45-1.25)			
rs6893892 C>T	---	C/C	123 (98.4)	281 (98.9)	1.00	0.15	542.4	767.2
		T/C	2 (1.6)	3 (1.1)	0.16 (0.01-2.13)			
r1494555 T>C	Recessive	T/T-C/T	99 (82.5)	237 (89.1)	1.00	0.13	522.3	739.9
		C/C	21 (17.5)	29 (10.9)	0.57 (0.28-1.17)			
rs2228141 C>T	Dominant	C/C	97 (80.8)	199 (71.6)	1.00	0.10	525.6	748.9
		T/C-T/T	23 (19.2)	79 (28.4)	1.64 (0.89-3.02)			
rs6897932 C>T	Dominant	C/C	77 (64.2)	192 (69.3)	1.00	0.57	523.5	742.6
		T/C-T/T	43 (35.8)	85 (30.7)	0.86 (0.51-1.45)			
rs987106 T>A	Recessive	T/T-T/A	93 (74.4)	199 (71.3)	1.00	0.21	542.4	766.5
		A/A	32 (25.6)	80 (28.7)	1.42 (0.81-2.47)			
rs3194051 A>G	Recessive	A/A-G/A	112 (94.1)	246 (88.8)	1.00	**0.04**	521.2	744.2
		G/G	7 (5.9)	31 (11.2)	2.51 (0.97-6.48)			
rs10491434 T>C	Recessive	T/T-T/C	108 (92.3)	246 (88.2)	1.00	0.16	522.3	745.3
		C/C	9 (7.7)	33 (11.8)	1.83 (0.77-4.33)			

Figure S1. Genetic study for *IL-7* gene variants. **A)** Summary of allele frequencies for *IL-7* single nucleotide polymorphisms (SNPs) in this study and in the Iberian Population in Spain (IBS) listed on the NCBI SNP database. **B)** Basic information of *IL-7* SNPs in the overall study cohort. **C**) *IL-7* SNP association with low pre-ART $CD4^+$ T-cell counts and also with poor immune recovery after 48 weeks of ART adjusted by age and baseline $CD4^+$ T-cell counts. Data analysis is summarized with n (%), odds ratio (OR) and 95% confidence interval (CI). The AIC and BIC values were used to choose the inheritance model (IHT) that best fits the data. HWE, Hardy-Weinberg equilibrium; AIC, Akaike's Information Criteria; BIC, Bayesian Information Criteria.

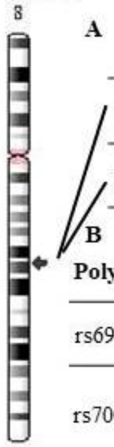

A

Polymorphism	Sequence	Chr Position	Typed samples	Allele	IBS	Controls	Cases	
							INR	IR
rs6987789 G>T	CRch38.p12	78740486	409/416 (98.3%)	G	1.00	0.99	0.99	0.99
				T	0.00	0.01	0.01	0.01
rs7007634 A>G	CRch38.p12	78807362	400/416 (96.2%)	A	0.90	0.89	0.89	0.93
				G	0.10	0.11	0.11	0.07

B

Polymorphism	Genotype	Cases		HWE	Controls		HWE	INR		HWE	IR		HWE
		Count	Proportion		Count	Proportion		Count	Proportion		Count	Proportion	
rs6987789 G>T	G/G	194	0.97	1	206	0.98	1	92	0.98	1	102	0.97	1
	G/T	5	0.03		4	0.02		2	0.02		3	0.03	
rs7007634 A>G	A/A	159	0.83	1	166	0.8	1	72	0.79	1	87	0.86	1
	A/G	32	0.17		40	0.19		18	0.20		14	0.14	
	G/G	1	0.01		2	0.01		1	0.01		0	0.00	

C

Polymorphism	IHT model	Genotype	Cases	Controls	OR (95% CI)	P-value	AIC	BIC
rs6987789 G>T	---	G/G	190 (97.4)	203 (98.1)	1.00	0.95	26.2	42.2
		T/G	5 (2.6)	4 (1.9)	NA (0.00-NA)			
rs7007634 A>G	Dominant	A/A	156 (83)	165 (80.5)	1.00	0.73	25.9	41.8
		G/A-G/G	32 (17)	40 (19.5)	0.44 (0.00-57.83)			

Polymorphism	IHT model	Genotype	INR	IR	OR (95% CI)	P-value	AIC	BIC
rs6987789 G>T	---	G/G	90 (97.8)	100 (97.1)	1.00	0.42	230.2	243.3
		T/G	2 (2.2)	3 (2.9)	2.32 (0.29-18.59)			
rs7007634 A>G	Dominant	A/A	71 (79.8)	85 (85.9)	1.00	0.52	223.6	236.5
		G/A-G/G	18 (20.2)	14 (14.1)	0.75 (0.31-1.82)			

Figure S2. Summary of allele frequencies for *IL-7R* single nucleotide polymorphisms (SNPs) in this study and in the Iberian Population in Spain (IBS) listed on the NCBI SNP database.

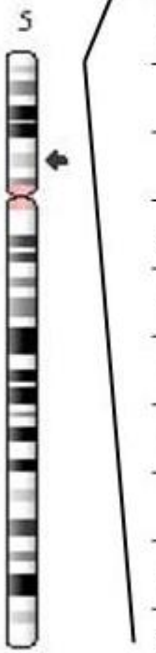

Polymorphism	Sequence	Chr Position	Typed samples	Allele	IBS	Controls	Cases	
							INR	IR
rs7701176 T>A	CRch38.p12	35856014	415/416 (99.8%)	T	0.99	0.99	0.99	0.99
				A	0.01	0.01	0.01	0.01
rs1494559 A>G	CRch38.p12	35860665	391/416 (93.9%)	A	0.85	0.86	0.89	0.84
				G	0.15	0.14	0.11	0.16
rs1494558 G>A	CRch38.p12	35860966	382/416 (91.8%)	G	0.65	0.67	0.60	0.70
				A	0.35	0.33	0.40	0.30
rs969128 A>G	CRch38.p12	35861057	386/416 (92.8%)	A	0.85	0.83	0.88	0.80
				G	0.15	0.17	0.12	0.20
rs969129 G>T	CRch38.p12	35861166	392/416 (94.2%)	G	0.66	0.68	0.62	0.71
				T	0.34	0.32	0.38	0.29
rs6893892 C>T	CRch38.p12	35870908	409/416 (98.3%)	C	1.00	0.99	0.99	1.00
				T	0.00	0.01	0.01	0.00
rs1494555 T>C	CRch38.p12	35871088	386/416 (92.8%)	T	0.66	0.67	0.60	0.71
				C	0.34	0.33	0.40	0.29
rs2228141 C>T	CRch38.p12	35871171	398/416 (95.7%)	C	0.85	0.86	0.90	0.83
				T	0.15	0.14	0.10	0.17
rs6897932 C>T	CRch38.p12	35874473	398/416 (95.4%)	C	0.75	0.80	0.84	0.84
				T	0.25	0.20	0.16	0.16
rs987106 T>A	CRch38.p12	35875491	404/416 (97.1%)	T	0.50	0.49	0.54	0.49
				A	0.50	0.51	0.46	0.51
rs3194051 A>G	CRch38.p12	35876172	396/416 (95.2%)	A	0.75	0.70	0.72	0.69
				G	0.25	0.30	0.28	0.31
rs10491434 T>C	CRch38.p12	35877812	396/416 (95.2%)	T	0.74	0.69	0.71	0.68
				C	0.26	0.31	0.29	0.32

Figure S3. Linkage disequilibrium analysis in cases (low pre-ART $CD4^+$ T-cell counts) versus controls for *IL-7R* gene variants.

rs7701176	*rs1494559*	*rs1494558*	*rs969128*	*rs969129*	*rs6893892*	*rs1494555*	*rs2228141*	*rs6897932*	*rs987106*	*rs3194051*	*rs10491434*
rs7701176	0.5391<br>0.1137<br>**0.0015**	0.9715<br>-0.0592<br>0.1018	0.5331<br>0.1016<br>**0.0048**	0.977<br>-0.058<br>0.1045	0.3909<br>0.3592<br>**0**	0.9777<br>-0.0594<br>**0.099**	0.5435<br>0.1164<br>**0.001**	0.9591<br>-0.0388<br>0.2743	0.3299<br>-0.0281<br>0.4248	0.6111<br>-0.0342<br>0.3367	0.0597<br>0.0077<br>0.829
	rs1494559	0.9987<br>-0.2885<br>**0**	0.9902<br>0.8945<br>**0**	0.9986<br>-0.2813<br>**0**	0.9065<br>-0.0288<br>0.4236	0.9987<br>-0.2879<br>**0**	0.9924<br>0.9771<br>**0**	0.8823<br>-0.1694<br>**0**	0.9449<br>-0.3814<br>**0**	0.9423<br>-0.2499<br>**0**	0.9432<br>-0.2531<br>**0**
		rs1494558	0.9987<br>-0.3194<br>**0**	0.99<br>0.9654<br>**0**	0.961<br>-0.0538<br>0.1389	0.9763<br>0.9743<br>**0**	0.9987<br>-0.2841<br>**0**	0.9993<br>-0.3387<br>**0**	0.9997<br>-0.7121<br>**0**	0.9996<br>-0.4678<br>**0**	0.9453<br>-0.4477<br>**0**
			rs969128	0.9986<br>-0.3115<br>**0**	0.9213<br>-0.0324<br>0.3723	0.9987<br>-0.3188<br>**0**	0.9906<br>0.8809<br>**0**	0.1383<br>-0.0294<br>0.4218	0.6114<br>-0.2732<br>**0**	0.9985<br>-0.2931<br>**0**	0.9985<br>-0.2967<br>**0**
				rs969129	0.9597<br>-0.0524<br>0.1454	0.9966<br>0.9738<br>**0**	0.9987<br>-0.277<br>**0**	0.9648<br>-0.3189<br>**0**	0.991<br>-0.6883<br>**0**	0.9996<br>-0.4562<br>**0**	0.9996<br>-0.4617<br>**0**
					rs6893892	0.9609<br>-0.0537<br>0.1388	0.9039<br>-0.0282<br>0.4286	0.0532<br>0.0088<br>0.8052	0.9738<br>0.0766<br>**0.031**	0.0021<br>2,00E-04<br>0.9945	0.9811<br>0.116<br>**0.0012**
						rs1494555	0.9987<br>-0.2835<br>**0**	0.9993<br>-0.338<br>**0**	0.9911<br>-0.7045<br>**0**	0.9996<br>-0.4669<br>**0**	0.9996<br>-0.4725<br>**0**
							rs2228141	0.9976<br>-0.1886<br>**0**	0.9991<br>-0.397<br>**0**	0.9985<br>-0.2607<br>**0**	0.9986<br>-0.2638<br>**0**
								rs6897932	0.9995<br>0.4756<br>**0**	0.9992<br>-0.3109<br>**0**	0.9992<br>-0.3146<br>**0**
									rs987106	0.9997<br>0.6569<br>**0**	0.959<br>0.6377<br>**0**
										rs3194051	0.9998<br>0.9879<br>**0**
											rs10491434

D'
r
P-value

P-value

- < 0.01 (red)
- 0.01 – 0.09 (orange)
- 0.10 – 0.50 (yellow)
- 0.51 – 1.00 (pale yellow)
